# Assessing pubertal status in multi-ethnic primary schoolchildren

**DOI:** 10.1111/apa.12850

**Published:** 2014-11-17

**Authors:** Sooky Lum, Vassiliki Bountziouka, Seeromanie Harding, Angie Wade, Simon Lee, Janet Stocks

**Affiliations:** 1 Respiratory, Critical Care & Anaesthesia Section (Portex Unit), UCL Institute of Child HealthLondon, UK; 2 Medical Research Council/Chief Scientist Office, Social and Public Health Sciences Unit, University of GlasgowGlasgow, UK; 3 Clinical Epidemiology, Nutrition and Biostatistics Section, UCL Institute of Child HealthLondon, UK

Pubertal status is related to childhood growth and independently associated with health outcomes such as lung function, blood pressure and mental health (1). However, self-assessment of pubertal status is difficult in young children as relevant questions may either be too difficult for young children, especially boys, to answer reliably or perceived to be culturally inappropriate (1–5). This is especially true for overweight children (6). The Size and Lung function In Children (SLIC) study was designed to explore ethnic differences in lung function and body physique in a multi-ethnic population of London schoolchildren (7). As part of this study, we collected both self-reports and parental reports of pubertal status in children aged eight to 11 years, both to investigate the feasibility of assessing the attainment of secondary sex characteristics, as a proxy for pubertal status in this population, and to explore any ethnic differences in rates of pubertal attainment.

## Methods

The SLIC study took place from 2011 to 2013 and comprised children aged 5–11 years, with parental consent, from 14 London schools with a high ethnic mix (7). A wide range of physiological and anthropometric baseline assessments were undertaken in the schools, with assessments being repeated a year later whenever possible. Parents provided information about their own ethnicity, and their child's, by filling in a study questionnaire. This was used to broadly categorise children as: White (European ancestry), of Black African origin (Black African or Black Caribbean descent); of south Asian origin (from India, Pakistan, Bangladesh or Sri Lanka) and other/mixed ethnicities. Socio-economic circumstances were measured using the Family Affluence Scale (7).

During the baseline assessments, children who were more than 8 years old were asked to complete a validated illustrated Tanner pubertal questionnaire (8) in private, supervised by investigators. Towards the end of the first year of baseline data collection, the Tanner questionnaire was withdrawn following concerns from one parent, as this could have potentially jeopardised the success of the whole study. The protocol was amended to include an additional question in the parental questionnaire for children over 8 years of age for implementation at the follow-up assessments 12 months later. This required a yes or no answer to the following question: ‘Has your child entered puberty (indicated by growth of armpit or pubic hair, and/or lowering of voice for boys or menstruation/periods for girls)?’ The study was guided by a steering committee and was approved by the London-Hampstead research ethics committee. Parental written consent and verbal assent from each child were obtained prior to assessments.

Data from children whose health status could impact on normal growth and pubertal development, such as those with congenital abnormalities, sickle cell disease or growth treatment, were excluded. Pubertal attainment was defined as having reached Tanner Stage 3 in their physical and, or, pubic hair development (8), during which a growth spurt typically occurs, as characterised by the most rapid linear growth and weight gain since infancy (9). Consequently, self-reports and parental reports of pubertal attainment were compared with height, weight and age. Logistic regression models were used to evaluate characteristics related to the adjusted odds of a child having reached puberty using R (Version 3.1.0; http://www.r-project.org). The significance level was set at 0.05.

**Figure 1:**
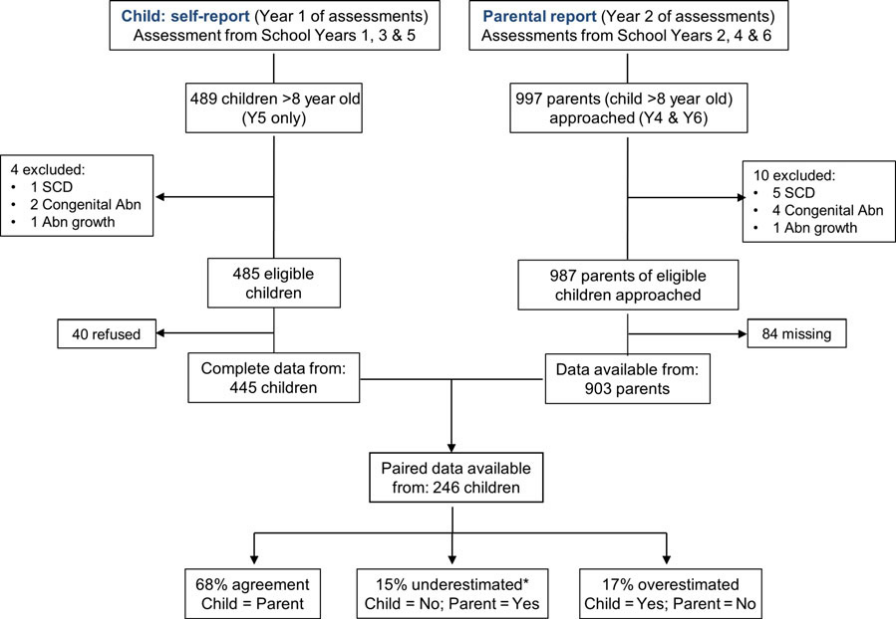
Study recruitment and assessment flowchart. Abn, abnormality; SCD, Sickle cell disease; Y, School year; yr, year. School year 1(Y1) equivalent to children aged 5–6 years; Y2: aged 6–7 years; Y3: 7–8 years; Y4: 8–9 years; Y5: 9–10 years; Y6: 10–11 years. *Information from both sources may be correct in that the child may not have attained puberty until a year later. Thus, the proportions of underestimation could be considerably <15%.

**Figure 2:**
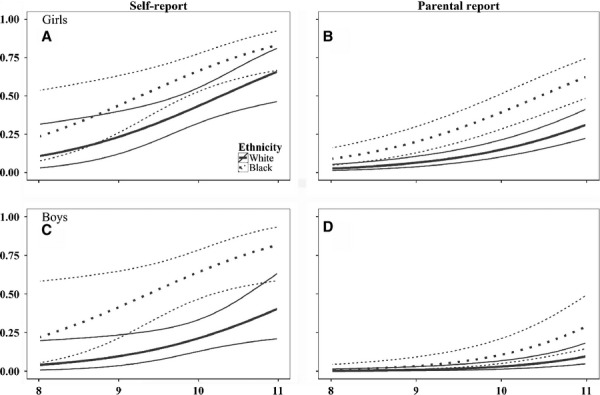
Probability of stating pubertal status attained according to self- (A & C) or parental report (B & D). The plot was derived from models presented in Table1 and has been simplified to Black African origin versus White children with mean z-Height and z-BMI for children of that age for illustrative purposes. The thick solid line with the accompanying thinner solid lines either side denote the mean (95% CI) predictive probability for White children to have reported that they have attained puberty at any given age while the dotted lines denote the predictive probabilities for children of Black African origin. When compared with predicted probabilities from parental reports, the children were more likely to say they had attained puberty at any given age. This difference was more marked in boys (C & D). Predicted probabilities for all ethnic groups are also available upon request.

Pubertal information from self-reports collected during baseline assessments was available from 445 of the 485 (92%) children aged 8–10 years and from 903 of the 987 (92%) parental reports for children aged 8–11 years at follow-up 1 year later (Fig.1 7). According to both the self-reports and the parental reports, missing pubertal data occurred more often for children of Black African origin (13%) than White (7%) children. Self-reported pubertal data were missing more often for children if their family spoke English as their first language (10% versus 5%), and parental reports were missing more often when English was not the dominant language at home (12% versus 6%) and in those from the lower, less advantaged Family Affluence Scale households (3% advantaged versus 15% least advantaged). When we looked at the self-reported pubertal questionnaires we received, 25% of the girls and 62% of the boys stated that they were ‘not sure’ when classifying some aspects of their pubertal development. On average, pubertal children were significantly older, taller and heavier than their non-pubertal peers, as reported previously (7), and this pattern was clearer from the parental than the children's reports. According to the parental reports, a higher proportion of children of Black African origin (16% of boys and 46% of girls) were reported to have attained puberty than the other ethnic groups combined (6% of boys and 23% of girls). After adjusting for age, height and body mass index (BMI) z-scores (10), children of Black African origin were more likely than their White counterparts to have attained puberty (Table1; Fig.2). Socio-economic circumstances were not associated with pubertal attainment from either source, regardless of gender (data not shown). The probability of reporting attainment of pubertal status according to self-reports versus parental reports is shown in Figure2. Paired data on pubertal status were available from 246 children, based on self-reports in year one and parental reports in year two, and the agreement between parental and child data were examined. These comprised 103 White children, 47 Black children 64 South Asian children and 32 of other and mixed ethnicities. Due to the low numbers with paired data in each ethnic group, the analysis was carried out as a single group. While there was agreement between parental reports and self-reports of pubertal status in at least 68% (95% CI, range 62–73%) of the children, attainment of puberty was overestimated by self-reports in 17% (13–22%) of the children and may have been underestimated by 15% (11–20%), although the latter is more difficult to ascertain as the child may have attained puberty by the time of the parental report a year later (Fig.1). Although this difference was non-significant, boys were, on average, more likely to overestimate pubertal status than girls of the same age, ethnicity and Family Affluence Scale with an odds ratio (95% CI) of 1.9 (0.93–3.7). As a result, we only used the pubertal data obtained from the parents for subsequent analysis.

**Table 1:** Factors associated with the adjusted odds of attaining puberty by self or parental assessment

	Self-report (n = 445) Odds ratio (95% CI)	Parental report (n = 903) Odds ratio (95% CI)		
	Girls (n = 268)	Boys (n = 177)	Girls (n = 506)	Boys (n = 397)
Age (per year)	3.1 (1.7; 5.8)	2.0 (0.99; 4.3)	2.6 (2.0; 3.3)	3.4 (2.1; 6.2)
Ethnicity (baseline: White)						
Black	2.2 (1.1; 4.5)	5.1 (2.2; 12)	3.7 (1.9; 7.2)	3.8 (1.3; 11)
South Asian	0.8 (0.3; 1.8)	0.6 (1.5; 1.8)	2.3 (1.1; 4.8)	1.4 (0.3; 5.3)
Other/mixed	0.7 (0.3; 1.5)	1.1 (0.4; 2.7)	1.9 (1.0; 3.4)	1.1 (0.3; 3.4)
z-Height* (per unit)	1.3 (1.0; 1.8)	0.9 (0.6; 1.2)	1.9 (1.5; 2.5)	1.4 (0.9; 2.2)
z-Body Mass Index* (per unit)	1.5 (1.2; 1.9)	1.1 (0.9; 1.5)	1.1 (0.9; 1.3)	1.5 (1.1; 2.2)

* Height and BMI were adjusted for age and sex and expressed as z (or SD score) using British 1990 reference (10).

The larger number of subjects assessed by parental than self-report reflects the fact that by the time of the 1-year follow-up when parental assessments were undertaken, a much higher proportion of the cohort was more than 8 years old and hence eligible for these assessments. The odds of a girl stating that she had attained puberty were 3.1 times higher for each year increase in the child's age, after taking ethnicity, z-Height and z-BMI into account. For example: the odds of an 11-year-old girl stating she had attained puberty were ∽9.6 (95% CI: 2.9; 33.6) times higher than those for a 9-year-old girl of the same ethnicity, z-Height and z-BMI.

It was feasible to collect information on pubertal status in children with self or parental reports, as 92% of the responses were received using these approaches. However, given that 25% of the girls and 62% of the boys were unsure of some aspects of their pubertal development, the reliability of the self-reports was questionable. While agreement between the self-reports and parental assessments was found in at least 68% of the pubertal assessments, we found that self-assessment was overestimated in 17% of the children, especially by younger children and boys, suggesting that for large epidemiological studies, parental assessments may be more reliable and less time-consuming. Given that somatic growth is highly correlated with pubertal development (9), with children categorised as having attained puberty being significantly older, taller and heavier than their non-pubertal peers, parental reports appeared to be more consistent than self-reports (Fig.2). The rate of pubertal attainment in 10- to 11-year-old girls of Black African or White European origin in this study was similar to that reported on the timing of menarche in a similar area of London based on teachers' assessment (11). Furthermore, our finding that children of Black African origin were more likely to have attained puberty at any of the ages studied than other ethnic groups is consistent with other reports (1,5,11), although a larger study spanning a wider age range is required to confirm these results in boys.

As all the assessments were undertaken in schools, traditional methods of determining pubertal status by physical examination were not deemed acceptable, particularly in the absence of parents, nor would this be feasible when undertaking large epidemiological studies (4). To date, the use of parental reports for assessing pubertal maturity in children is limited. Carskadon and Acebo found good correlations between self-reports and parental assessments in children aged 10–12 years (12). Although the Avon Longitudinal Study of Parents and Children did assess pubertal status using self-reports and parental reports, details as to which data sets were used in their analyses, or the extent to which results from the two approaches agreed, were not reported (13,14). To assist compliance and minimise risk of missing data, we only included one concise question in the parental questionnaire regarding pubertal markers. While secondary sex characteristics prior to adult reproductive function and onset of the menarche are clearly defined in girls, pubertal development in boys is less clear without physical examination (9). As a result, voice change was included as a complementary pubertal marker for boys (9,15). The high response from the parental questionnaires demonstrated that this approach may be more reliable and acceptable than self-assessment, especially for children under the age of 12. Pubertal data from self-reports and parental reports were collected 12 months apart, which limits the accuracy of interpretation. Menarche and voice breaking are both very late signs of puberty, and a child will have had some development for at least a year before these occur. The pattern of puberty differs between boys and girls. The pubertal growth spurt starts when breast development occurs in girls, but is delayed until mid-puberty in boys. Boys are likely to have some pubertal development, such as hair and testicular growth, which no one other than parents would notice prior to their pubertal growth spurt.

These findings suggest that parental reports of pubertal development may be preferable for large epidemiological studies when crude estimates of maturation are needed for children under 12 years of age, as these provide greater certainty than self-reports by children of this age.
